# Beyond the Bile: Exploring the Microbiome and Metabolites in Cholangiocarcinoma

**DOI:** 10.3390/life14060698

**Published:** 2024-05-29

**Authors:** Jungnam Lee, Hanul Kim, Jin-Seok Park

**Affiliations:** 1 Department of Internal Medicine, Inha University Hospital, Inha University School of Medicine, Incheon 22332, Republic of Korea; jungnamlee@inha.ac.kr (J.L.); khu0830@gmail.com (H.K.); 2 Department of Internal Medicine, Shihwa Medical Center, Siheung 15034, Republic of Korea

**Keywords:** cholangiocarcinoma, microbiome, metabolite, 16S rRNA gene sequencing

## Abstract

Introduction: Cholangiocarcinoma (CCC) still has a high mortality rate despite improvements in diagnostic and therapeutic techniques. The role of the human microbiome in CCC is poorly understood, and a recent metagenomic analysis demonstrated a significant correlation between microbiome-associated carcinogenesis and CCC. This study aimed to investigate changes in microbiome composition associated with CCC and its metabolic signature by integrating taxonomic and functional information with metabolomics data and in vitro experimental results. Methods: From February 2019 to January 2021, this study included patients who underwent endoscopic retrograde cholangiopancreatography (ERCP), both with and without a diagnosis of CCC. Bile samples were collected via endoscopic nasobiliary drainages (ENBD) and subjected to DNA extraction, PCR amplification of the bacterial 16S rRNA gene V3-V4 region, and data analysis using QIIME2. In vitro Carboxyfluorescein succinimidyl ester (CFSE) proliferation and Annexin V/PI apoptosis assays were performed to investigate the effects of metabolites on CCC cells. Results: A total of 24 patients were included in the study. Bile fluid analysis revealed a significantly higher abundance of *Escherichia coli* in the CCC group. Alpha diversity analyses exhibited significant differences between the CCC and non-CCC groups, and Nuclear Magnetic Resonance (NMR) spectroscopy metabolic profiling identified 15 metabolites with significant concentration differences; isoleucine showed the most notable difference. In vitro experiments demonstrated that isoleucine suppressed CCC cell proliferation but did not induce apoptosis. Conclusions: This research underlines the significance of biliary dysbiosis and specific bile metabolites, such as isoleucine, in influencing the development and progression of CCC.

## 1. Introduction

Cholangiocarcinoma (CCC) is an epithelial neoplasm that originates from different regions within the biliary tract and exhibits characteristics indicative of cholangiocyte differentiation [1,2]. Being the second most prevalent primary liver cancer following hepatocellular carcinoma, CCC constitutes approximately 15% of all primary liver tumors and 3% of gastrointestinal neoplasms [2]. Despite diagnostic and therapeutic improvements, CCC still exhibits a high mortality rate because it is often asymptomatic during the early stages. This leads to diagnosis in advanced stages, severely limiting therapeutic options, and resulting in a poor prognosis [1,3,4]. Surgical resection remains the optimal and exclusive curative approach [5]. Nevertheless, the high rate of recurrence following surgical intervention significantly prevents optimal clinical outcomes. In addition, a substantial proportion of patients present with unresectable tumors upon initial diagnosis, which limits their therapeutic choices and further exacerbates disease progress [5,6].

Despite extensive research, the underlying pathogenesis of CCC remains poorly understood. While chronic inflammation caused by exposure to liver flukes or primary sclerosing cholangitis is considered a major contributing factor, the specific role played by human microbiota in the carcinogenesis of CCC remains unclear [3,7]. Bile juice used to be considered sterile [8], but recent investigations, including ours, have revealed the presence of a complex bile microbiome in individuals with and without biliary diseases [9,10,11,12]. Furthermore, metagenomic analysis of these bile samples has demonstrated a significant correlation between CCC and carcinogenesis associated with microbiota [13,14,15]. Metabolic profiles or metabolomes reflect microbiome activity and can be quantitatively determined using mass spectrometry. We believe that the integration of metabolomic data with microbiome taxonomic composition may enable the identification of a metabolic signature with potential applications in the prevention or early diagnosis of CCC.

Bile metabolites, the byproducts of metabolic activities in the liver and biliary tract, are crucial for the digestion and absorption of nutrients. Changes in the profiles of these metabolites are noted in various liver diseases, including cirrhosis, cholestasis, and hepatocellular carcinoma. Given their influence on hepatobiliary health, it is imperative to explore their roles in the development of CCC and their interactions with the biliary microbiome [3,14].

Therefore, we conducted 16S rRNA gene sequencing analyses of bile samples obtained from patients with or without CCC to explore the possible roles of the biliary microbiome and bile metabolites during CCC development, with the goal of uncovering novel diagnostic and therapeutic strategies.

## 2. Methods

### 2.1. Patient Selection and Comparative Analysis of Bile Microbiome

We performed a comprehensive bile fluid microbiome investigation in individuals who underwent endoscopic retrograde cholangiopancreatography (ERCP) at our institution between February 2019 and January 2021. The objective was to identify the microbiome differences between patients with CCC and the controls. Eligible patients were ≥18 years old and had undergone ERCP for bile duct decompression. CCC diagnosis was confirmed via computed tomography (CT) or magnetic resonance imaging (MRI) scans. Patients were excluded if they had hemolytic anemia, severe liver disease, or presented with challenging endoscopic approaches due to esophageal or duodenal stenosis, gastric outlet obstruction, or a medical history associated with thrombocytopenia or coagulopathy (platelet count ≤ 60,000/mm^3^ or PT-INR ≥ 1.5; normal PT-INR range, 0.85–1.25).

### 2.2. Bile Fluid Collection during ERCP

ERCP was conducted on selected patients using a conventional side-viewing duodenoscope (TJF-260; Olympus Corporation, Tokyo, Japan) along with a regular straight injection catheter. Once the therapeutic goals of the ERCP were achieved, an endoscopic nasobiliary drainage tube (ENBD) was carefully placed in the proximal common bile duct (CBD). To mitigate the risk of bile contamination from the upper gastrointestinal system, including the oral cavity, bile samples (20–30 cc) were collected from the CBD through ENBDs 24 h post-procedure. These samples were quickly placed into sterile sputum containers and stored at −80 °C until analysis.

### 2.3. Extraction of DNA from Bile Samples

Bile samples were centrifuged at 5000× *g* for five minutes at room temperature. They were then suspended in 500 μL of cetyltrimethyl ammonium bromide buffer as per the manufacturer’s guidelines. DNA extraction was carried out using the Maxwell^®^ RSC PureFood GMO and Authentication Kit (Promega, Madison, WI, USA) following the manufacturer’s guidelines. The quantification of bacterial DNA was performed with a UV-vis spectrophotometer (NanoDrop 2000c; Thermo Fisher Scientific, Waltham, MA, USA) and the QuantiFluor^®^ ONE dsDNA System (Promega). Prior to shipment for DNA analysis and sequencing at Bioeleven Co., Ltd. (Seoul, Republic of Korea), bile specimens were preserved at −80 °C. Specimens subjected to PCR were maintained at −20 °C until required.

### 2.4. Amplification of the V3–V4 Variable Region of the Bacterial 16S rRNA Gene

Two-step PCR was used to amplify the V3–V4 variable region of the bacterial 16S rRNA gene. In brief, two primers were used, a forward primer (5′-TCGTCGGCAGCGTCAGATGTGTATAAGAGACAGCCTACG-GGNGGCWGCAG) and a reverse primer (5′-GTCTCGTGGGCTCGGAGATGTGTATAAGAGACA-GGACTACHVGGGTATC-TAATCC-3′). The PCR products underwent agarose gel electrophoresis in a 2% gel, and the 16S rRNA libraries were subsequently cleaned up using magnetic beads (AMPure XP) following the manufacturer’s protocols (Beckman Coulter, Wycombe, UK). The purity of the samples was verified using a Bioanalyzer 2100 (Agilent, Santa Clara, CA, USA). During the second-round PCR phase, Illumina Nextera barcodes (Illumina, Inc., San Diego, CA, USA) were added to the initial PCR products using i5 and i7 primers. The amplified products were purified as described in the initial PCR. DNA quantification was performed with the QuantiFluor^®^ ONE dsDNA System (Promega), and the Bioanalyzer 2100 was utilized for quality assessment of the samples. The 16S rRNA genes were amplified and a library was prepared using the two-step PCR protocol. 16S rRNA sequencing was carried out using the MiSeq v3 Reagent Kit (Illumina, Inc.).

### 2.5. Data Analysis

16S rRNA sequencing information was analyzed using the QIIME2 pipeline (version 2022.11) [16]. Low-quality base reads (quality score < 30) were filtered and trimmed using Trimmomatic v0.39 [17]. Following this, the DNA sequences were organized into amplicon sequence variants (ASVs) through reference-based clustering, utilizing the Greengenes rRNA database (Release 138) [18]. Species richness and differences in microbial profiles were assessed based on alpha and beta diversity indices computed with QIIME2. To assess alpha diversity, microbial diversity was evaluated using ASV richness. Three indices were used to appraise alpha diversity: observed ASVs, Dominance D, and the Shannon index. Beta diversity is a metric of compositional dissimilarity between ASVs in phylogenetic trees. Bray–Curtis distance matrices generated from metagenomes, predicted using QIIME, were subjected to analyses of similarities (ANOSIM).

### 2.6. Nuclear Magnetic Resonance Spectroscopy (NMR) Metabolomics

Bile samples were filtered through pre-washed 3 kDa spin filters (NANOSEP 3 K, Pall Life Sciences) at 10,000× *g* for 2–3 h at 4 °C. Filtrates were mixed with nuclear magnetic resonance spectroscopy (NMR) buffer containing 100 mM phosphate buffer in D2O (pH 7.3) and 1.0 mM 3-Trimethylsilyl 2,2,3,3-d4 propionate (TMSP); the final sample volume was 600 μL, and the TMSP concentration was 0.5 mM. The experiments were conducted on 550 μL samples in 103.5 mm × 5 mm NMR tubes (Bruker Analytik, Rheinstetten, Germany). One-dimensional 1H-NOESY NMR spectra, along with two-dimensional data, 1H-1H total correlation spectroscopy, and 1H-13C heteronuclear single quantum coherence data, were acquired using a Bruker Avance II 600 MHz spectrometer that operates with Topspin 3.6 software). Chemical shifts were assigned to metabolites based on the reference spectra in the Human Metabolome Database and the Chenomx NMR Suite profiling software database (Chenomx Inc., Edmonton, AB, Canada, version 8.4). A total of 41 metabolites were quantified using the Chenomx software with TMSP as the internal standard. Statistical analyses were performed using R-studio and MetaboAnalyst 5.0.

### 2.7. Cell Culture

SNU-1196 CCC cells were purchased from the Korean Cell Line Bank (Seoul, Republic of Korea). Cells were cultured in the RPMI 1640 medium supplemented with 10% FBS (Gibco^TM^, Carlsbad, CA, USA), 2 mM glutamine, and 100 U/mL penicillin–streptomycin (Gibco^TM^), and maintained in a 5% CO_2_ incubator set at a temperature of 37 °C.

### 2.8. CFSE Proliferation Assay

To determine whether isoleucine inhibits CCC cell proliferation, SNU-1196 cells were cultured in 24-well plates at a density of 10^6^ cells/well and treated with 1, 10, or 50 mM L-isoleucine (SLCH6428, Sigma-Aldrich, St. Louis, MO, USA). Cells were pulse-labeled through incubation with 2.5 μM Carboxyfluorescein succinimidyl ester (CFSE, C34570, ThermoFisher Scientific) in 0.5% FBS RPMI 1640 medium for 20 min and cultured for 72 h. During this growth period, the cells were collected at 24, 48, and 72 h and subjected to flow cytometric analysis using CytoFLEX (Beckman Coulter, Brea, CA, USA).

### 2.9. Annexin V/PI Apoptosis Assay

An Annexin V-FITC apoptosis detection kit (#550475, BD Biosciences Pharmingen, USA) was used to quantify apoptotic/necrotic cells. This kit allows for the distinction between early apoptotic cells (annexin V-positive and propidium iodide (PI)-negative), necrotic cells (positive for both annexin V and PI), and viable cells (negative for both annexin V and PI). Cell counts were determined via flow cytometry with a CytoFLEX unit (Beckman Coulter, USA).

### 2.10. Ethics Statement

The Institutional Review Board of Inha University Hospital approved the study protocol on July 15, 2019 (INHAUH 201902015). Informed written consent was obtained from all participants prior to the commencement of the study. Additionally, the study adhered to the ethical guidelines outlined in the Declaration of Helsinki.

## 3. Results

### 3.1. Characteristics of Patients

Twenty-four patients were enrolled in the study. Of these, 11 patients underwent ERCP for CCC group, while the other 13 (non-CCC group) underwent ERCP for the management of benign tumors (n = 1), benign biliary strictures (n = 1), CBD stones (n = 4), CBD bile sludge (n = 2), dilated bile ducts (n = 4), and autoimmune pancreatitis (n = 1). Patients’ baseline characteristics are summarized in Table 1.

### 3.2. Bile Microbiome Taxonomic Analysis

**Figure 1:**
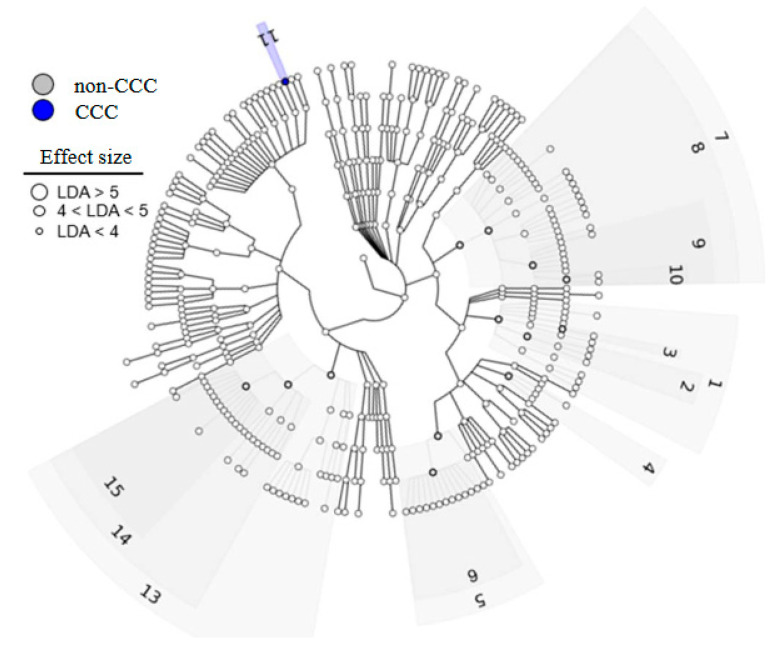
Cladogram showing significantly different abundances of bacterial taxa.

**Figure 2:**
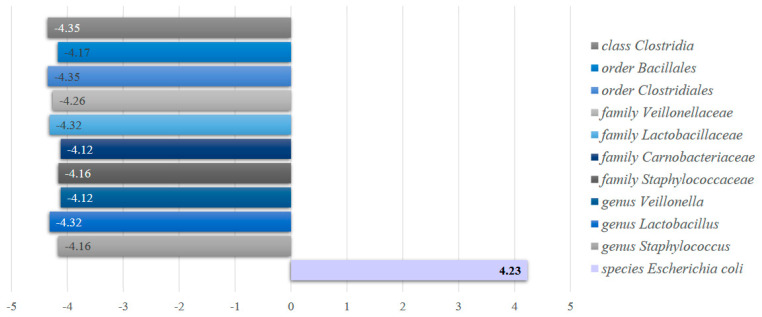
LDA scores of bacterial taxa.

Linear discriminant analysis effect size (LEfSe) was used to identify major differences between the biliary microbiomes of the CCC and non-CCC groups. Linear discriminant analysis (LDA) revealed that *Escherichia coli* (LDA score = 4.23) was significantly more abundant in the CCC group (Figure 1). In addition, the *Clostridia* class, *Bacillales* and *Clostridiales* orders, *Veillonellaceae*, *Lactobacillaceae*, *Carnobacteriaceae*, and *Staphylococcaceae* families, and *Veillonella*, *Lactobacillus*, and *Staphylococcus* genera were significantly more abundant in the non-CCC group (Figure 2).

### 3.3. Alpha and Beta Diversities

**Figure 3:**
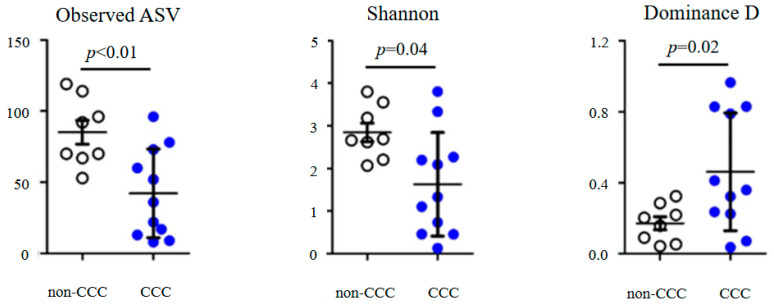
Alpha diversities (observed ASVs, Shannon and dominance D); empty circles represent the non-CCC samples and blue circles represent the CCC samples.

The alpha diversity analysis revealed significant differences between the CCC and non-CCC groups (Figure 3). The observed ASVs (*p* < 0.01) and Shannon (*p* = 0.04) diversities were more abundant in the non-CCC group. However, the dominance D index (*p* = 0.02), which quantifies the prevalence of one or more species within a community, was higher in the CCC group. This suggests that bile samples from the CCC group had lower microbial diversity compared to those from the non-CCC group.

**Figure 4:**
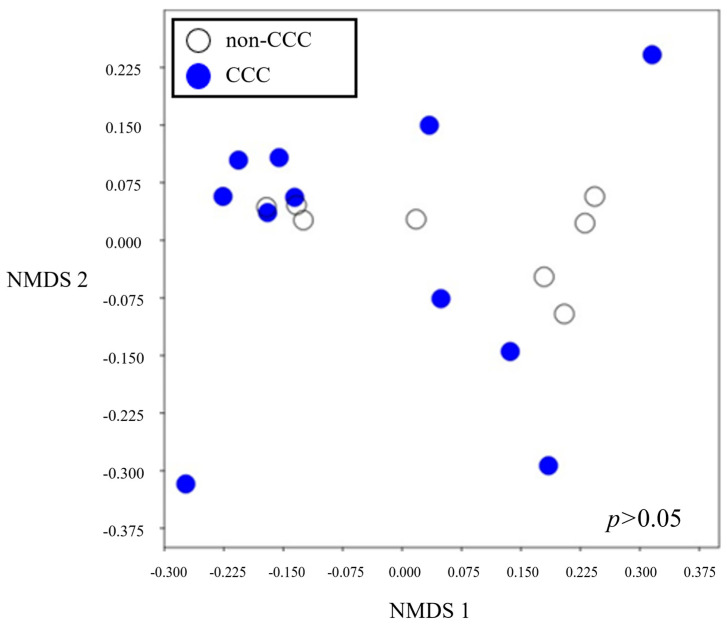
Beta diversity. Results of group beta diversity analysis were determined using Bray–Curtis distance matrices and the ANOSIM method.

Beta diversity provides a valuable means of quantifying differences or similarities between microbial compositions across groups (Figure 4). Interestingly, beta diversity revealed similarities between the gut microbiomes of the two groups (*p* > 0.05).

### 3.4. Metabolic Profiles of Bile Fluid as Determined by NMR

**Figure 5:**
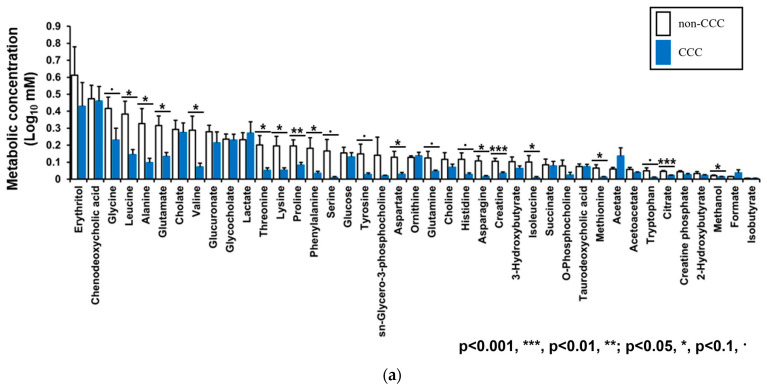

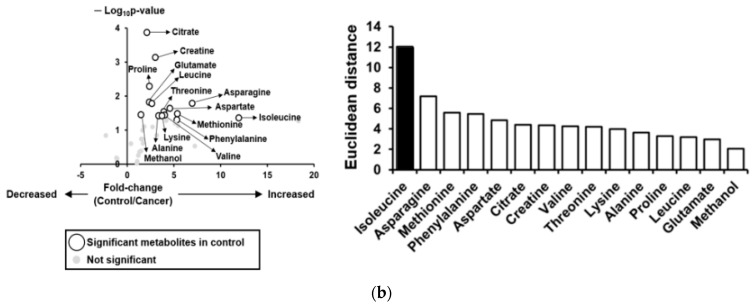
NMR spectroscopy. (**a**) Forty-one metabolites were identified by NMR spectroscopy. (**b**) Isoleucine concentrations showed the greatest difference between the bile samples of CCC and control patients.

In total, 41 metabolites were detected by NMR, and the abundances of 15 were significantly different between the two groups. Interestingly, all significant metabolites were more concentrated in the bile samples from the non-CCC group (Figure 5a), with isoleucine showing the largest concentration difference (Figure 5b).

### 3.5. In Vitro Experiment

**Figure 6:**
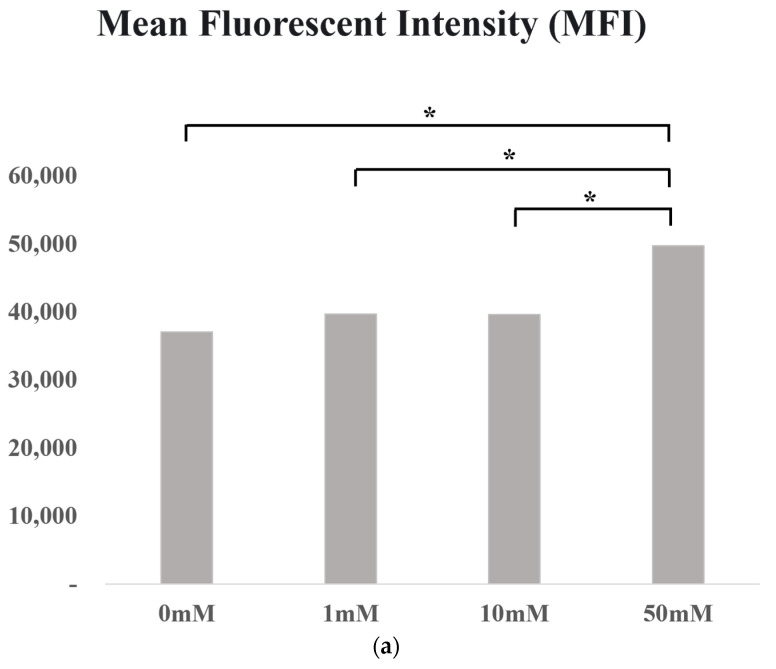

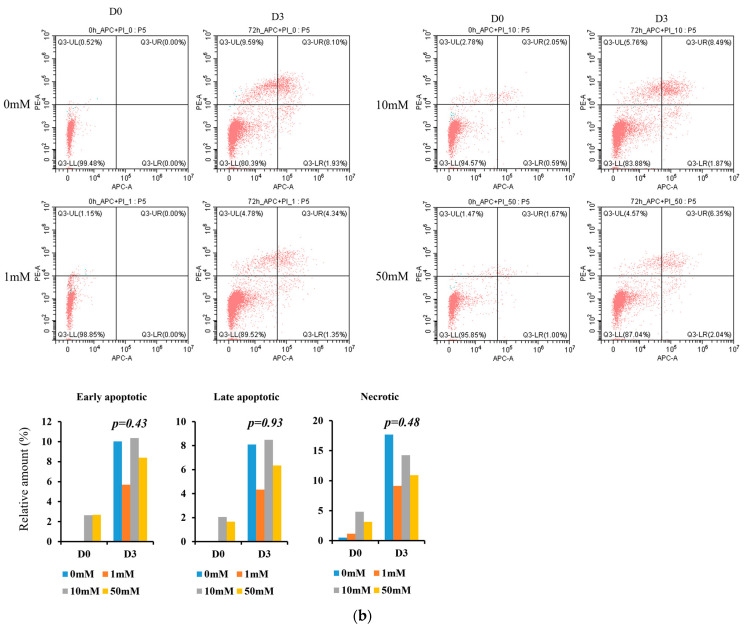
In vitro experiment; (**a**) CFSE proliferation assay, indicated by an asterisk (*), which signifies *p* < 0.05, denoting statistical significance; (**b**) Annexin V/PI apoptosis assay.

The effect of isoleucine on SNU-1196 cell proliferation was quantitatively analyzed using the CFSE assay. During the 3-day culture period, cancer cells treated with isoleucine exhibited significantly slower proliferation rates compared to untreated cells. Notably, the inhibitory effect of isoleucine on cell proliferation was prominent at a high concentration of 50 mM. In contrast, lower concentrations of 1 mM and 10 mM did not result in statistically significant changes in proliferation rates. These findings are depicted in Figure 6a, which illustrates the mean fluorescent intensity (MFI) indicative of cell proliferation across different treatment groups. Annexin V/PI staining showed that isoleucine treatment did not lead to a significant increase in the percentage of early or late apoptotic or necrotic cells, with *p*-values of 0.43, 0.93, and 0.48, respectively. These findings suggest that the antitumor effect of isoleucine was due to the suppression of cancer cell proliferation rather than the induction of cell death (Figure 6b).

## 4. Discussion

In this study, we investigated the roles of microbial dysbiosis and bile metabolites in CCC development. Our comparative analysis of the biliary microbiomes of patients with and without CCC revealed significant differences, indicating microbial dysbiosis in patients with CCC. Specifically, patients with CCC had a higher *Escherichia coli* abundance at the species level. Additionally, we analyzed bile metabolic profiles to identify potential CCC biomarkers. Our findings indicate that the concentrations of several metabolites, especially isoleucine, were significantly lower in patients with CCC. In vitro experiments investigating the effect of isoleucine on CCC showed that its antitumor effect was primarily due to the suppression of proliferation rather than the induction of apoptosis. These findings suggest that dysbiosis of the biliary microbiome and alterations in bile metabolites such as increased isoleucine levels may contribute to the development and progression of CCC. The microbiome plays a critical role in regulating amino acid metabolism, which has been implicated in cancer development. Alterations in the gut microbiome can lead to dysregulated amino acid metabolism, which can have various tumorigenic effects, such as the promotion of inflammation, cell proliferation and survival, and the alteration of cellular signaling pathways [19,20,21,22,23,24]. Specifically, changes in the gut microbiota have been linked to imbalances in the levels of certain amino acids, such as tryptophan, methionine, and serine, which can contribute to the development of colorectal, liver, and gastric cancers [14,23,25]. Our study showed that the bile samples of patients with CCC contained less diverse microbial communities than those of non-cancer patients. Furthermore, an analysis of the CCC microbiome revealed *Escherichia coli* as the predominant species. *Escherichia coli* synthesizes isoleucine from threonine and homoserine through a series of enzymatic reactions and catabolizes isoleucine for energy production [26]. Isoleucine is an essential amino acid involved in various biological processes, including protein synthesis and energy metabolism [19,27]. Previous studies have shown that alterations in the gut microbiome, including changes in the abundance of *Escherichia coli*, can lead to the dysregulation of amino acid metabolism and contribute to the development of various diseases, including cancer.

We found that isoleucine levels in bile were lower in patients with CCC than those in the controls. However, the role of isoleucine in cancer development is controversial; therefore, we investigated the role of isoleucine in CCC. Interestingly, our in vitro experiments demonstrated that isoleucine has an antitumor effect, and that this is due to the suppression of proliferation rather than the induction of apoptosis. These findings suggest that isoleucine may play a role in the pathogenesis of CCC and is a potential biomarker for early detection and therapeutic intervention. Remarkably, a previous study on lung cancer reported similar results regarding the effects of isoleucine on cancer cell proliferation. Specifically, high doses of isoleucine stabilized nuclear PTEN and thus inhibited lung cancer cell proliferation [27]. While the precise mechanism underlying the effect of isoleucine on CCC cells remains unclear, these findings suggest that it may have similar effects on cancer cells in different contexts. Further studies using xenograft models or other approaches are required to investigate the effects of isoleucine on CCC development, and to elucidate the mechanisms underlying its antitumor effect.

Our study has several notable strengths. Most importantly, we used CCC bile samples, which are not readily accessible, to analyze the biliary microbiome and metabolites, and compared these results with those of patients without cancer. This approach provides a unique perspective on the potential role of dysbiosis in CCC development. In addition, we conducted cellular experiments to demonstrate the biological relevance of our findings, and to provide a proof of concept. Specifically, our in vitro experiments showed that isoleucine suppresses CCC cell proliferation. However, this study also has several limitations. First, the sample size was small. Thus, our results should be validated with larger cohorts. Second, although we conducted cellular experiments to investigate the biological relevance of our results, further in vivo studies are necessary to confirm our observations and assess their potential clinical applications. Third, we only investigated the biliary microbiome and metabolites; other factors such as host genetics and environmental exposure may also play a role in the development and progression of CCC.

Despite limitations, this study provides valuable insights into the potential roles of biliary microbiome dysbiosis and alterations in bile metabolites, particularly isoleucine, in the development and progression of CCC. These insights can potentially guide the development of new diagnostic and therapeutic strategies for this challenging disease. However, future studies must adopt a more comprehensive approach to elucidate the mechanisms underlying CCC development.

## Figures and Tables

**Table 1:** Demographic profiles of the study subjects.

Variables	Total (n = 24)	Cholangiocarcinoma (n = 11)	Non-Cholangiocarcinoma (n = 13)	* p-Value
Age (years) ^§^	73 (42–88)	75 (58–88)	72 (42–84)	0.69
Sex, male, n (%)	17 (70.8)	8 (72.7)	9 (69.2)	0.61
Hypertension, n (%)	12 (50)	4 (36.4)	8 (61.5)	0.41
DM, n (%)	4 (16.7)	3 (27.3)	1 (7.7)	0.30
Dyslipidemia, n (%)	10 (41.7)	6 (54.5)	4 (30.8)	0.41
WBC (/uL) ^§^	9618 (3640–29,130)	7428 (3640–14,720)	11,471 (5410–29,130)	0.12
CRP, mg/dL ^§^	3.9 (0.1–28.7)	4.3 (0.1–16.8)	3.5 (0.1–28.7)	0.77
T.bil, mg/dL ^§^	5.9 (0.2–27.9)	8.7 (0.3–27.9)	3.5 (0.2–23.9)	0.12
AST, IU/L ^§^	167.6 (19.0–663.0)	206.3 (23.0–625.0)	134.8 (19.0–663.0)	0.40
ALT, IU/L ^§^	136.3 (10.0–688.0)	131.1 (14.0–380.0)	140.8 (10.0–688.0)	0.89
ALP, IU/L ^§^	424.1 (45.0–2781.0)	742.8 (109.0–2781.0)	154.4 (45.0–630.0)	0.05

Abbreviations: WBC, white blood cell count; CRP, C-reactive protein; T.bil, total bilirubin; AST, alanine aspartate transferase; ALT, alanine aminotransferase; ALP, alkaline phosphatase; ^§^, median (range); *, *p* values were calculated using the *t*-test or the Chi-square test.

## Data Availability

The raw data are available from the corresponding author upon receipt of a reasonable request and have been deposited in the publicly accessible NCBI database.

## Abbreviations

CCC, cholangiocarcinoma; ERCP, endoscopic retrograde cholangiopancreatography; CT, computed tomography; MRI, magnetic resonance imaging; ENBD, endoscopic nasobiliary drainage; CBD, common bile duct; ASVs, amplicon sequence variants; NMR, nuclear magnetic resonance spectroscopy; CFSE, carboxyfluorescein succinimidyl ester; LEfSe, linear discriminant analysis effect sizes; LDA, linear discriminant analysis; MFI, mean fluorescent intensity
